# Association of Knee Osteoarthritis Treatment Types, Patient Characteristics, and Medical History With Subsequent Risk for Total Knee Arthroplasty: Data From a New Real-World Registry

**DOI:** 10.1016/j.artd.2025.101643

**Published:** 2025-02-26

**Authors:** Andrew L. Concoff, Jennifer H. Lin, Andrew I. Spitzer, Vinod Dasa, Adam Rivadeneyra, David Rogenmoser, Mitchell K. Ng, Mary DiGiorgi, Stan Dysart, Joshua Urban, William M. Mihalko, Michael A. Mont

**Affiliations:** a Exagen Inc., Vista, CA, USA; b Pacira BioSciences, Inc., Tampa, FL, USA; c Department of Orthopaedic Surgery, Cedars Sinai Medical Center, Los Angeles, CA, USA; d Department of Orthopaedic Surgery, Louisiana State University Health Services Center, New Orleans, LA, USA; e Orthopaedic Specialty Institute, Orange, CA, USA; f Mid State Orthopaedic & Sports Medicine Center, Alexandria, LA, USA; g Department of Orthopaedic Surgery, Maimonides Medical Center, Brooklyn, NY, USA; h OrthoNebraska, Omaha, NE, USA; i Department of Orthopaedic Surgery, University of Tennessee Health Science Center, Campbell Clinic Orthopaedics, Memphis, TN, USA; j Department of Orthopaedic Surgery, Sinai Hospital of Baltimore, Baltimore, MD, USA

**Keywords:** Total knee arthroplasty, Non-operative, Pain, Triamcinolone extended-release, Cryoneurolysis

## Abstract

**Background:**

This article examines predictors of subsequent total knee arthroplasty (TKA) within 6 months of nonoperative intervention based on (1) patient demographics; (2) knee osteoarthritis (OA) severity; and (3) various nonoperative treatments (cryoneurolysis with superficial or deep genicular nerve block, intra-articular [IA] hyaluronic acid injections, nonsteroidal inflammatory drug injections, IA-corticosteroids injections, or IA-triamcinolone extended-release [IA-TA-ER] injections).

**Methods:**

Patients who had unilateral knee OA and received nonoperative intervention were identified in the Innovations in Genicular Outcomes Research registry between September 2021 and February 2024, identifying 505 patients. Baseline patient demographics were tabulated by knee OA severity as graded by Kellgren-Lawrence (KL) and nonoperative treatment, identifying patients who underwent TKA within 6 months. Predictors of TKA were identified using 20 potential demographic/clinical variables and calculating individual hazard ratios.

**Results:**

Obesity and KL grade IV knees were significant predictors of TKA within 6 months of nonoperative treatment (*P* < .05). Age, sex, marital status, number of comorbidities, physical activity level, smoking status, insurance type, and baseline pain and functional scores were not associated with subsequent TKA. Overall, treatment type was also not linked to subsequent TKA, although pairwise comparison suggested use of IA-TA-ER was associated with a decreased conversion to subsequent TKA (*P* = .002).

**Conclusions:**

Apart from obesity and KL grade IV knees, it remains challenging to identify which patients are at risk for conversion to subsequent TKA after nonoperative treatment. It appears IA hyaluronic acid and IA-TA-ER are most associated with decreased conversion to TKA within 6 months.

## Introduction

Total knee arthroplasty (TKA) continues to be the definitive treatment for end-stage knee osteoarthritis (OA) that does not respond to nonsurgical treatments, with approximately 800,000 procedures performed annually in the United States [1]. Although primary TKA is a relatively safe and effective method for managing end-stage knee OA, there are numerous treatment alternatives that could potentially postpone or eliminate the need for TKA. Broadly, available treatments for knee OA can be categorized into intra-articular (IA) injections and denervation-based therapies [1]. IA injections include IA corticosteroids (IA-CS) [2], IA hyaluronic acid (IA-HA) compounds, extended-release corticosteroid formulations such as triamcinolone acetonide, and cellular-based therapies, which primarily target synovial inflammation and pain by suppressing collagenases, aggrecans, and other proinflammatory mediators [[3], [4], [5]]. Denervation-based treatments, such as cryo-neurolysis and radiofrequency ablation techniques [6], focus on interrupting pain signals from the knee. Cryoneurolysis, specifically, applies extreme cold temperatures (between −20°C and −100°C) to peripheral nerves, causing Wallerian degeneration and temporary disruption of nerve function to reduce pain [7].

They enable prospective study designs, standardized data collection, and the inclusion of patient-reported outcomes along with other clinical and health utilization metrics [[8], [9], [10], [11], [12]]. The Innovations in Genicular Outcomes Research (iGOR) registry, for instance, employs a prospective observational study design to systematically gather data on the clinical efficacy, safety, patient quality of life, and overall healthcare utilization associated with various knee OA treatments [13,14].

While there is much literature regarding TKA outcomes and the safety and efficacy of various nonoperative treatment modalities for symptomatic knee OA, there is limited work that has examined specific predictors of subsequent TKA after nonoperative management. This article aimed to identify predictors of subsequent TKA within 6 months of nonoperative intervention based on (1) patient demographics; (2) knee OA severity; and (3) 6 nonoperative therapies (cryoneurolysis with deep genicular nerve block [Cryo-Deep/Both], cryoneurolysis of the superficial nerve block [Cryo-Superficial], IA-HA injections, nonsteroidal anti-inflammatory drug [IA-NSAID] injections, IA-CS injections, or IA-triamcinolone extended-release [IA-TA-ER] injections). We hypothesized that obesity, higher Kellgren-Lawrence (KL) grades, and certain nonoperative treatments would be associated with an increased likelihood of subsequent TKA within 6 months.

## Material and methods

### Registry

The iGOR registry, recorded on Clinicaltrials.gov with the identifier NCT05495334, is an active, multicenter, prospective, longitudinal observational registry designed to monitor clinical and health-related outcomes for up to 18 months after various knee OA pain interventions [14]. As a purely observational registry, iGOR leaves all clinical decision-making to the treating physicians, mirroring real clinical practice [13]. It documents a broad spectrum of OA treatments, including oral medications like nonsteroidal anti-inflammatory drugs (NSAIDs) and opioids; denervation procedures such as radiofrequency ablation and cryoneurolysis; IA injections including corticosteroids, viscosupplementation, stem cell products, platelet-rich plasma, and NSAIDs; and surgical treatments like arthroplasty. Currently, participants have been recruited from 8 diverse sites, including academic medical centers and outpatient clinics, as follows: Campbell Clinic, Memphis, TN; Cedars Sinai Medical Center, Los Angeles, CA; Hoag Orthopedic Institute, Orange, CA; Mid State Orthopedics, Alexandria, LA; LSU/Ochsner Medical Center, New Orleans, LA; Sinai Hospital of Baltimore, Baltimore, MD; and United Rheumatology, Hauppauge, NY, with plans to expand the registry to a total of 11 locations throughout the United States. Funding for this study and the iGOR database creation and maintenance was provided by Pacira (Pacira BioSciences, Parsippany, New Jersey), with data collected on various nonoperative knee OA treatments. Approval was secured from an Institutional Review Board at each site with research support (Pacira BioSciences, Parsippany, New Jersey) in compliance with the International Conference on Harmonization, Good Clinical Practice guidelines, and the US Food and Drug Administration Title 21 Code of Federal Regulations Part 56.

Participants eligible for the iGOR registry include any patients scheduled to undergo nonoperative treatment for knee OA within 60 days of screening, which may include injections, nerve blocks, or arthroplasty, provided they can offer informed consent. Additionally, previous patients undergoing further treatment are continuously screened by research coordinators at each site and are followed in the registry for another 18 months, starting from their latest treatment episode. Patients were excluded if they were currently participating in a clinical trial that restricted standard care interventions, received more than 1 treatment type, or those scheduled for surgery unrelated to the treated knee.

### Patient cohort

We identified patients who have knee OA who were receiving nonsurgical treatments: cryoneurolysis of the deep genicular nerve (Cryo-Deep/Both), cryoneurolysis of the superficial genicular nerve (Cryo-Superficial), IA-HA injections, IA-NSAIDs, IA-CS, and IA-TA-ER. The Cryo-Deep/Both and Cryo-Superficial treatments use the Iovera device (Pacira BioSciences, Parsippany, New Jersey). This handheld device delivers extremely cold temperatures to peripheral nerve tissues, potentially blocking or alleviating pain for up to 3 months [15,16]. Cryoneurolysis is applied using either a 20-gauge, 90-mm closed-end needle or 3 27-gauge, 8-mm closed-end needles, allowing targeted exposure to low temperatures. This is achieved through the flow of cryogen (nitrous oxide) from a cartridge through a handpiece to a SmartTip (Pacira Cryotech, Inc., Fremont, California).

**Figure 1:**
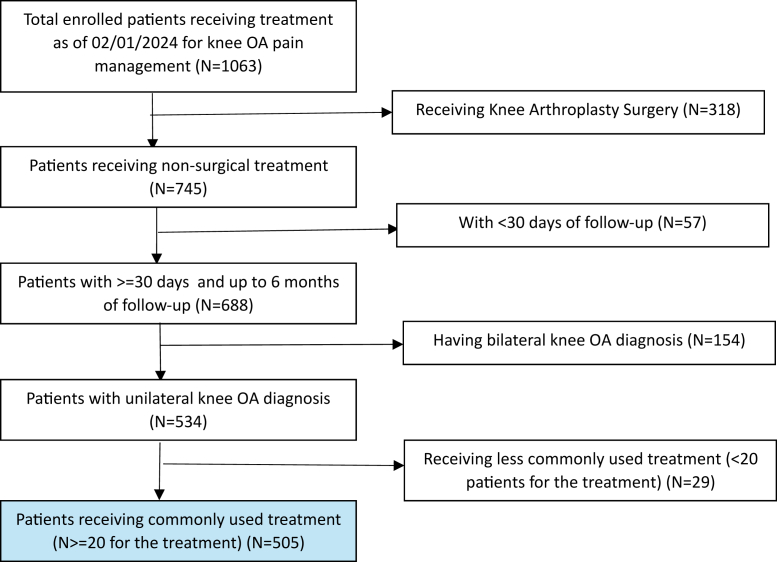
The iGOR study population. iGOR, Innovations in Genicular Outcomes Research; OA, osteoarthritis.

ZILRETTA (32 mg) (Pacira BioSciences, Parsippany, New Jersey), an extended-release injectable suspension of triamcinolone acetonide, was the chosen product for IA-TA-ER treatments [[17], [18], [19]]. The specific types of other IA injections, including NSAIDs, conventional corticosteroids, and hyaluronic acid, were selected based on the healthcare provider’s judgment. A total of 505 patients who had symptomatic unilateral knee OA and received 1 type of initial nonsurgical treatment were enrolled between September 21, 2021 and February 1, 2024, with at least 30 days and up to 6 months of follow-up (Fig. 1). Baseline patient demographics were collected, including age, sex, race, body mass index (BMI), medical history, surgical history, OA treatment history, smoking status, physical activity level, and prior analgesic medication history (Table 1).

**Table 1:** Distribution of patient characteristics.

Patient characteristics	Patient (N = 505)
Follow-up period after treatment (in days), mean (standard deviation, SD)	109 (59)
Age, mean (SD), year	63 (10)
Sex, number (%)	
Women	366 (72)
Men	139 (28)
Race, number (%)	
White	310 (61)
Black or African American	155 (31)
Asian	11 (2)
Other/unknown	29 (6)
Marital status, number (%)	
Single	199 (39)
Married	305 (61)
Veteran status, number (%)	
Active	18 (4)
Discharged or not a veteran	415 (82)
Missing	72 (14)
Educational level, number (%)	
High school or lower	120 (24)
College or higher	385 (76)
Insurance type, number (%)	
Commercial	186 (37)
Medicaid	61 (12)
Medicare	244 (48)
Other	14 (3)
Employment status, number (%)	
Currently employed	232 (46)
Currently unemployed/retired/other	273 (54)
Physical activity level, number (%)	
Sedentary	30 (6)
Light	284 (56)
Moderate	157 (31)
Vigorous	34 (7)
Body mass index (BMI), mean (SD), kg/m^2^	35 (11)
Kellgren-Lawrence grade, number (%)	
1 (doubtful)	17 (3)
2 (mild)	101 (20)
3 (moderate)	170 (34)
4 (severe)	204 (40)
Missing	13 (3)
Number of comorbidities, number (%)	
0	128 (25)
1-2	316 (63)
≥3	61 (12)
Prior opioid use, number (%)	
Yes	80 (16)
No	425 (84)
Index nonsurgical treatment, number (%)	
Cryoneurolysis_both/deep	71 (14)
IA-HA	78 (15)
IA-NSAID	45 (9)
IA-CS	216 (43)
Cryoneurolysis_superficial	23 (6)
IA-TA-ER	72 (14)
Baseline pain catastrophizing scale score, mean (SD)	
Baseline BPI pain severity score, mean (SD)	23 (14)
Baseline KOOS, JR functional score, mean (SD)	5 (2)
Baseline PROMIS, sleep disturbance (SD)	47 (17)
Follow-up healthcare facility visit prior to TKA, mean (SD)	55 (9)

KOOS, knee injury and osteoarthritis outcome score; BPI, brief pain inventory; IA-CS, intra-articular corticosteroids; IA-NSAID, intra-articular nonsteroidal anti-inflammatory drug; IA-TA-ER, intra-articular triamcinolone extended-release; IA-HA, intra-articular hyaluronic acid; KOOS, JR, knee injury and osteoarthritis outcome score, joint replacement; PROMIS, patient-reported outcomes measurement information system; SD, standard deviation; TKA, total knee arthroplasty.

### Predictors of subsequent total knee arthroplasty

A series of demographic variables were preselected and assessed to see if they were associated with subsequent primary TKA during the 6 months of follow-up during treatment. These variables can be broadly categorized into patient demographics, severity of knee OA, and various nonoperative treatments for knee OA. The severity of knee OA was assessed through the KL grade graded by individual research sites’ attendings and clinical coordinators, and a radiographic classification of OA from 1 to 4, including joint space narrowing, osteophytes, subchondral sclerosis, and deformity of bony ends (category: 1-4) [20].

The number of healthcare facility visits (eg, emergency room, hospital, outpatient clinic) was recorded following treatment and before TKA or end of study period (continuous: ranged from 0 and more), whichever occurred first. The number of patients in each category was quantified along with the subsequent number undergoing TKA in the 6-month follow-up, with a hazard ratio generated to identify risk factors for subsequent TKA.

### Data analyses

Data analysis was conducted using Statistical Analysis Software Version 9.4 (SAS Institute, Cary, North Carolina). Descriptive statistics were used to summarize baseline demographics and measurements collected during follow-up across different study groups. A priori power analysis was conducted to determine the sample size required to detect a significant hazard ratio for the predictors of interest. Assuming an alpha of 0.05, a power of 0.80, and an expected hazard ratio of 1.5 for significant predictors, a minimum of 500 patients were required. Our sample size of 505 patients meets this requirement. Multivariate Cox proportional hazard regression modeling was performed to identify any plausible predictors for subsequent TKA during the 6 months of follow-up following treatment, with each corresponding variable as previously listed. Statistical significance was set at a cutoff *P* value of .05. Corrections for multiple testing (ie, 20 tests) were provided in the section on sensitivity analysis (new cutoff *P* value: .0025).

## Results

### Baseline characteristics

A total of 505 patients who received 1 of the 6 nonsurgical treatments for symptomatic knee OA in iGOR at study enrollment and who had up to 6 months of treatment follow-up (average: 109 days) were included in the present analysis (Table 1), of whom 24 progressed to primary TKA (4.8%). There were 43% of patients receiving IA-CS (N = 216), followed by 15% of patients receiving IA-HA (N = 78), 14% of patients receiving IA-TA-ER (N = 72), or Cryo-Deep/Both (N = 71). Less than 10% of patients received IA-NSAIDs (9%, N = 45) and cryoneurolysis of the superficial genicular nerve (6%, N = 23). Overall, patients had an average age of 63 years, with 72% being women. The majority of patients were obese (mean BMI: 35; standard deviation: 11) and had moderate to severe KL grades III or IV (74%).

### Obesity associated with subsequent TKA

Obesity was a significant predictor of TKA within 6 months of nonoperative treatment (hazard ratio: 0.93; 95% confidence interval [CI]: 0.93-0.99; *P* = .03). Other baseline patient demographics were not associated with subsequent TKA within 6 months of nonoperative treatment with IA injections or cryoneurolysis (*P* > .05 for all), including age, sex, marital status, number of comorbidities, employment status, veteran status, physical activity level, smoking status, educational level, race, insurance type, baseline opioid use, baseline pain catastrophizing score (PCS), baseline functional score, baseline sleep disturbance score, and number of healthcare facility visits (Table 2).

**Table 2:** Association of 20 predictors with subsequent total knee arthroplasty during 6-month follow-up^a^.

Predictors	Level	N, total patients	N, TKA	Hazard ratio	95% CI		*P* value
					Lower	Upper	
Age		495	24	0.97	0.91	1.05	.483
Sex	Women	361	15	0.52	0.18	1.48	.219
	Men (ref)	134	9	1.00			
Kellgren-Lawrence grade							.033
	1	17	1	0.08	0.01	0.83	.034
	2	100	3	0.15	0.03	0.74	.020
	3	169	6	0.18	0.05	0.62	.007
	4 (ref)	196	14	1.00			
Marital status	Single	196	7	1.18	0.34	4.08	.795
	Married (ref)	299	17	1.00			
Number of comorbidities^b^				0.78	0.42	1.43	.419
Employment status	Employed	226	11	2.13	0.66	6.92	.208
	Unemployed/retired	269	13	1.00			
Veteran status	Active	17	1	0.42	0.04	5.06	.494
	Discharged or not a veteran (ref)	407	18	1.00			
Index nonsurgical treatment							.051
	Cryo-both/deep	71	10	18.65	2.96	117.42	.002
	IA-HA	78	0	0.00	0.00		.993
	IA-NSAID	43	2	14.13	0.87	230.74	.063
	IA-CS	212	8	5.27	0.86	32.52	.073
	Cryo-superficial	21	2	9.43	0.74	119.93	.084
	IA-TA-ER (ref)	70	2	1.00			
Physical activity level							.828
	Sedentary	30	1	1.00			
	Light	279	15	0.81	0.06	10.42	.871
	Moderate	153	7	0.48	0.03	7.05	.591
	Vigorous	33	1	0.41	0.01	14.36	.623
Smoking							.248
	Never	355	20	8.13	0.49	136.06	.145
	Past	97	3	3.91	0.19	79.10	.375
	Current (ref)	43	1	1.00			
Education level	Secondary or less	117	7	0.82	0.26	2.60	.733
	College or higher (ref)	378	17	1.00			
Race							.054
	White	306	21	20.49	1.63	258.35	
	Asian	11	0	0.00	0.00		
	Other/unknown	27	2	67.45	3.40	1338.4	
	Black (ref)	151	1	1.00			
Insurance type							.329
	Medicare (ref)	242	16	1.00			
	Medicaid	60	2	1.35	0.08	24.30	.837
	Commercial	180	5	0.27	0.05	1.34	.108
	Other	13	1	0.99	0.10	10.02	.996
Baseline opioid use	No (ref)	417	21	1.00			
	Yes	78	3	0.64	0.14	2.92	.569
BMI		495	24	0.93	0.87	0.99	.038
Baseline pain catastrophizing scale		495	24	0.96	0.92	1.02	.162
Baseline pain severity score		495	24	1.11	0.81	1.53	.526
Baseline functional score		495	24	0.97	0.92	1.02	.207
Baseline sleep disturbance score		495	24	0.98	0.92	1.05	.506
Number of healthcare facility visits^c^		495	24	1.05	0.73	1.51	.797

BMI, body mass index; CI, confidence interval; IA-CS, intra-articular corticosteroids; IA-NSAID, intra-articular nonsteroidal anti-inflammatory drug; IA-TA-ER, intra-articular triamcinolone extended-release; IA-HA, intra-articular hyaluronic acid; ref, reference; TKA, total knee arthroplasty.

a N total analyzed patients: 495; 10 patients who had a missing BMI were not included.

b Including cardiovascular disease (eg, chronic heart failure, peripheral vascular disease, coronary artery disease, myocardial infarction, hypertension, and venous thromboembolism), autoimmune disease, cancer, chronic liver disease, chronic lung disease, chronic renal disease, diabetes mellitus, and thyroid disease.

c Including outpatient clinic visits, ED visits, and inpatient admissions for up to 6-month follow-up since index treatment and prior to TKA.

### Kellgren-Lawrence IV knees associated with subsequent TKA relative to lower grades

Patients who have KL grade IV knees had a significant conversion to primary TKA within 6 months of nonoperative treatment relative to lower KL grades (*P =* .033). Patients who have KL grade I knees were associated with a 92% lower converstion to TKA (hazard ratio: 0.08; 95% CI: 0.01-0.83; *P* = .034), while those who have KL grade II knees were associated with an 85% lower conversion to primary TKA (hazard ratio: 0.15; 95% CI: 0.03-0.74; *P* = .02). Patients who have KL grade III knees had a lower conversion to TKA relative to those who have KL grade IV knees (hazard ratio: 0.018; 95% CI: 0.05-0.62; *P* = .007) (Table 2).

### IA-TA-ER is associated with a decreased conversion to TKA relative to deep cryoneurolysis

Overall, index nonsurgical treatment was not a major predictor of conversion to primary TKA (*P* > .05). Patients treated with IA-HA were free of subsequent TKA risk. For other treatment types, administration of IA-TA-ER was associated with a decreased conversion to subsequent TKA relative to Cryo-Deep/Both (hazard ratio: 18.65; 95% CI: 2.96-117.42; *P* = .002) (Table 2).

### Sensitivity analyses

Additional analysis performed by studying the potential interaction between BMI and KL grade was not statistically significant (hazard ratio: 0.96; 95% CI: 0.89-1.03; *P* = .219). When the analysis was restricted to patients who had a KL grade of 4 (40% of the total), the overall results remained similar, although the associations were attenuated (Table 3). Specifically, risk associations with treatment types and BMI were no longer statistically significant (*P* ≥ .24).

**Table 3:** Association of predictors with total knee arthroplasty risk in patients with severe knee osteoarthritis (ie, Kellgren-Lawrence grade = 4).

Predictors	Level	N, total patients	N, TKA	Hazard ratio	95% CI		*P* value
					Lower	Upper	
Age		196	14	1.01	0.91	1.13	.845
Sex	Women	143	9	0.89	0.18	4.45	.886
	Men (ref)	53	5	1.00			
Marital status	Single	87	5	1.07	0.20	5.84	.937
	Married (ref)	109	9	1.00			
Number of comorbidities^a^		196	14	0.39	0.13	1.17	.094
Employment status	Employed	81	5	0.69	0.09	5.30	.720
	Unemployed/retired	115	9	1.00			
Veteran status	Active	10	1	0.97	0.06	16.65	.983
	Discharged or not a veteran (ref)	162	12	1.00			
Index nonsurgical treatment							.240
	Cryo-both/deep	18	3	12.30	1.25	120.84	.031
	IA-HA	22	0	0.00	0.00		
	IA-NSAID	33	2	18.86	0.60	592.09	.095
	IA-CS	72	5	4.54	0.47	44.17	.192
	Cryo-superficial	14	2	37.03	1.00	1370.7	.050
	IA-TA-ER (ref)	37	2	1.00			
Physical activity level							.443
	Sedentary	14	1	1.40	0.04	48.44	.853
	Light	105	8	1.00			
	Moderate	63	4	0.24	0.04	1.44	.119
	Vigorous	14	1	0.36	0.01	10.27	.553
Smoking							.648
	Never	144	11	2.33	0.12	44.70	.575
	Past	36	2	0.98	0.03	30.43	.988
	Current (ref)	16	1	1.00			
Education level	Secondary or less	43	3	1.85	0.21	16.65	.585
	College or higher (ref)	153	11	1.00			
Race							.055
	White	130	11	13.28	0.54	328.57	.114
	Asian	4	0	0	0		
	Other/unknown	53	1	198.89	3.80	10,418.5	.009
	Black (ref)	9	2	1.00			
Insurance type							.850
	Medicare (ref)	99	9	1.00			
	Medicaid	29	2	3.01	0.10	87.81	.522
	Commercial	63	3	0.85	0.07	10.18	.895
	Other	5	0	0.00	0.00		
Baseline opioid use	No (ref)	160	12	1.00			
	Yes	36	2	1.50	0.17	13.71	.717
Body mass index				0.94	0.84	1.05	.272
Baseline PCS		196	14	0.97	0.87	1.07	.498
Baseline pain severity score		196	14	0.92	0.53	1.61	.772
Baseline functional score		196	14	0.97	0.90	1.05	.492
Baseline sleep disturbance score		196	14	0.95	0.86	1.05	.355
Number of healthcare facility visits^b^		196	14	0.92	0.48	1.78	.808

CI, confidence interval; IA-CS, intra-articular corticosteroids; IA-HA, intra-articular hyaluronic acid; IA-NSAID, intra-articular nonsteroidal anti-inflammatory drug; IA-TA-ER, intra-articular triamcinolone extended-release; PCS, pain catastrophizing score; ref, reference; TKA, total knee arthroplasty.

a Including cardiovascular disease (eg, chronic heart failure, peripheral vascular disease, coronary artery disease, myocardial infarction, hypertension, venous thromboembolism), autoimmune disease, cancer, chronic liver disease, chronic lung disease, chronic renal disease, diabetes mellitus, and thyroid disease.

b Including outpatient clinic visits, ED visits, and inpatient admissions for up to 6-month follow-up since index treatment and prior to TKA.

Also, the application of Bonferroni corrections showed that associations with most predictors were no longer statistically significant, except that the conversion to TKA after IA-TA-ER was still statistically significantly less than after Cryo-Deep/Both.

## Discussion

This study sought to assess 20 potential predictors for undergoing TKA within 6 months following nonoperative treatments, analyzing factors such as patient demographics, the severity of knee OA, and the type of nonoperative intervention. Our data demonstrated that obesity and KL grade IV knee conditions were linked to a higher likelihood of subsequent TKA before correction for multiple testing. However, other demographic factors like age, sex, marital status, comorbidity count, employment, veteran status, physical activity, smoking, education level, race, insurance type, initial opioid use, initial PCS, initial functional score, initial sleep disturbance score, and healthcare facility visit frequency showed no such association. Additionally, as a whole, the type of nonoperative treatment generally did not influence the likelihood of undergoing TKA. However, subgroup analysis revealed that IA extended-release triamcinolone injections were associated with a lower rate of subsequent TKA compared to Cryo-Deep/Both at 6 months

Among patient demographics, our study identified obesity as a risk factor for subsequent progression to TKA. Obesity is a well-known risk factor for the development of OA in weight-bearing joints, including the hip and knees, through both a direct increase in joint loading and an indirect effect on metabolic disturbances. Being an extra 10 pounds overweight amplifies the joint pressure on the knees by 30-40 pounds per step, which translates to a commensurate 36% increased risk of developing OA [21]. A study by Bourne *et al.* using the Canadian Joint Replacement Registry of 54,406 patients undergoing either primary total hip or knee arthroplasty demonstrated that the risk for TKA was 3.2 times higher for overweight individuals, 8.53 times higher for those who have obesity, and 18.78 times higher for patients who have morbid obesity [22]. Our study adds a temporal component to this information, as obese patients were at a higher subsequent conversion from nonoperative treatment to primary TKA within 6 months.

However, identifying other predictors of TKA was challenging. Our study found no significant association among age, sex, marital status, comorbidity count, employment, veteran status, physical activity, smoking, education level, race, insurance type, initial opioid use, initial PCS, initial functional score, initial sleep disturbance score, and subsequent primary TKA. Previous studies have attempted to examine risk factors for patients undergoing TKA [[23], [24], [25], [26]]. Women are overall more likely than men to develop arthritis in their knees [23] and often seek treatment at a later stage than men, who have greater functional disability at the time of surgery [24]. Our study demonstrated that in the immediate 6-month time period after index treatment with injection or cryoneurolysis, men were no different from women in the likelihood of undergoing a primary TKA. Current literature indicates that patients who are White are more likely to undergo a TKA than those who are Black. A retrospective study of 1070 patients who had symptomatic knee OA demonstrated that Black participants were significantly less likely to undergo primary TKA than their White counterparts (11% vs 19%, *P* < .001) [25]. A prognostic prediction model for TKA, including 2658 participants from the Multicenter Osteoarthritis Study and Osteoarthritis Initiative, found that KL grade, White race, and older age were most predictive of the conversion to TKA [26]. To the best of our knowledge, no study has looked at the number of healthcare visits as a risk factor. Our data suggest that the number of healthcare facility visits (outpatient clinic visits) is also not a factor for conversion to subsequent TKA. Overall, apart from obesity, the lack of association between these variables may be temporal-based. While prior studies may have indicated that White race and older age were most predictive of the lifetime conversion to TKA, our research indicates that in the 6 months following nonoperative intervention, this risk is not increased.

When considering knee OA radiographic severity, our results indicate that patients who have KL grade IV knees are associated with an increased conversion to TKA within 6 months following nonoperative treatment. One retrospective review of 207 patients who have symptomatic knee OA revealed that patients who have KL grade IV OA (odds ratio: 20.8; *P* = .009) and varus alignment (odds ratio: 13.0, *P* = .04) are risk factors for undergoing TKA and for decreased time from initial presentation to surgery [27]. The current key indication for TKA remains end-stage degenerative joint disease of the knee with persistent pain unresponsive to conservative treatment measures [28]. To this end, the natural history of symptomatic knee OA remains predominantly conservative. In an observational study of 167 patients who have symptomatic knee OA, 67% of patients had not undergone surgery within 6 years of the initial consultation. Of note, this study by Dabare et al. also demonstrated that patients who have KL grade IV OA were more likely to undergo surgery in a quicker time period [29]. Our data are consistent with these studies and provide additional information that patients who have KL grade IV knee OA are more likely to undergo TKA within 6 months.

Apart from nonmodifiable factors such as patient demographics and the severity of knee OA, potentially modifiable factors include the type of nonoperative treatment. Our data indicate that the overall type of treatment (cryo-superficial, cryo-deep, IA-HA injections, IA-NSAIDs, IA-CS, or IA-TA-ER) was not associated with subsequent TKA. A host of studies have examined the safety and efficacy of various nonoperative treatment options for symptomatic knee OA [[1], [3],^13^,[30], [31], [32], [33], [34]]. The goal of such interventions remains to improve pain or function and delay or prevent the need for a TKA. Prior literature has revealed evidence that IA-TA-ER is associated with decreased pain and improved function relative to other injections [13,35,36]. On subgroup analysis, our data suggest that IA-TA-ER is also associated with a decreased conversion to subsequent TKA relative to Cryo-Deep/Both. IA-TA-ER is synthesized to serve as an extended-release corticosteroid injection using microspheres comprised of 75:25 lactic to glycolic acid and poly lactic-co-glycolic acid approximately 45 microns in diameter [37] that allow for steady and slow drug elution over time [18]. The potential efficacy of IA-TA-ER has been established in several randomized clinical trials [35,36,38,39], all of which demonstrated significant pain relief relative to controls. However, to our knowledge, there are no significant data comparing the functional benefits of IA-TA-ER relative to other noninjection treatment modalities, such as cryoneurolysis. Our data suggest that, in addition to pain relief, IA-TA-ER is associated with decreased conversion to TKA at 6 months relative to Cryo-Deep/Both. Nevertheless, such an association was substantially attenuated among patients who had the most severe knee OA. To our knowledge, most patients treated with cryoneurolysis in the registry have failed most other nonsurgical treatments, suggesting that these patients likely have longer and more severe symptoms that are not available in the registry (eg, joint effusion, articular cartilage lesions). Patient choice (eg, nonsurgical treatment preference) may also play an important role, as indicated in our previous studies [40,41]. Our current analysis is preliminary and warrants confirmation in future larger studies.

This study is not without potential limitations inherent to its design. The iGOR registry established and maintained by Pacira BioSciences lacks a standard treatment protocol, leading to nonsystematic organization and distribution of patients into treatment categories. This randomness can result in variations in how patients receive treatments, influenced by differing treatment approaches and indications across multiple sites and medical specialties [13]. Furthermore, the variable numbers of patients can complicate the planning required for analytical comparisons among the treatments and may serve as an area for future potential study [42,43]. The short follow-up period of 6 months may not be sufficient to fully assess the long-term outcomes of nonoperative treatments and their influence on the progression to TKA. Particularly for treatments like IA-TA-ER, which are designed to provide relief for up to 6 months, patients may not opt for TKA within this period even if the treatment is not entirely effective. Additionally, the mean follow-up period was 109 days, which is less than the typical 90-day delay surgeons often observe between injections and TKA. This could introduce a bias where patients who might have proceeded to TKA after the study period are not accounted for, potentially underestimating the true rate of conversion to TKA. Second, there was a lack of standardization regarding the types and dosages of NSAIDs, steroid injections, and viscosupplementation injections used across different sites. This variability can affect the generalizability of the results and makes it challenging to draw definitive conclusions about the efficacy of specific treatments. Third, the observational design of the iGOR registry and its reliance on real-world clinical practices may introduce selection bias and confounding factors that are difficult to control for. For instance, the decision to proceed to TKA may be influenced by patient preferences, surgeon recommendations, or other unmeasured factors such as duration of symptoms before enrollment, which were not consistently captured in the registry. Furthermore, the funding and maintenance of the iGOR registry by Pacira BioSciences, the manufacturer of IA-TA-ER, could introduce potential conflicts of interest despite efforts to maintain objectivity. Operational management of the registry is conducted by individual sites, ranging from academic institutions to private clinical practices, without oversight from a Contract Research Organization. This setup can lead to a selection bias driven by the availability of staff and the allocation of resources for data collection. Employing technology in data collection broadens and eases participation but tends to skew the participant sample toward individuals who have higher socio-economic status and education levels and who are also tech-savvy. Despite these limitations, the overall implementation of the iGOR registry brings major benefits. It stands out as a collaborative, progressive real-world observational registry, excelling in gathering extensive data that reflect actual clinical practice and capture a wide range of treatments for knee OA, including patient-reported outcomes and clinical effectiveness.

One should also realize that a major potential limitation of this study is certainly a patient’s candidacy for a TKA, based on their medical condition as well as their individual as well as the physician’s choice. If the clinician views the patient as an appropriate candidate for TKA, then the assessment of the nonoperative treatment outcome is conditional upon being favorable enough to obviate the need for TKA. If the patient is not viewed by the clinician as an appropriate candidate for TKA when the nonoperative treatment choice is made [because either index knee damage is too mild (eg, by KL grade 1 to 3) or the severity of comorbid conditions such as deep venous thrombosis or pulmonary embolus risk, atherosclerotic coronary artery disease risk, or end-stage liver or kidney disease risks render the operative morbidity or mortality too high], then the nonoperative treatment outcome is assessed without consideration of the alternative of a TKA as an option. Thus, in analyzing whether a given nonoperative intervention is effective enough to convince a patient who should otherwise consider undergoing a TKA, not to do so, excluding those deemed inappropriate for TKA, more clearly defines an accurate denominator. Therefore, this confounds the results of the intended analysis to determine the relative impact of each nonoperative intervention on the likelihood and timeframe of subsequent TKA. Thus, in future analyses, we will generate larger numbers that are robust enough to allow for stratification by both candidacy in general and by baseline KL grade.

## Conclusions

This article aimed to identify 20 potential predictors of subsequent TKA within 6 months of nonoperative intervention based on patient demographics, knee OA severity, and various nonoperative treatments (cryoneurolysis of the superficial genicular nerve, Cryo-Deep/Both, IA-HA injections, nonsteroidal inflammatory drug injections, IA-CS, or IA-TA-ER injections). While patients who have obesity or KL grade IV knees were associated with subsequent TKA, other baseline patient demographics were not associated with subsequent TKA (age, sex, marital status, number of comorbidities, employment status, veteran status, physical activity level, smoking status, educational level, race, insurance type, baseline opioid use, baseline PCS, baseline functional score, baseline sleep disturbance score, and number of healthcare facility visits). Overall, the type of nonoperative treatment modality was not associated with subsequent TKA. However, on subgroup analysis, IA-TA-ER injections were associated with a decreased rate of subsequent TKA relative to Cryo-Deep/Both at 6 months; the data are preliminary and warrant further confirmation in larger studies with additional indicators for knee OA severity. The short follow-up period and potential biases limit the conclusiveness of these results. Further studies with longer follow-up periods, standardized treatment protocols, and comprehensive data collection are needed to confirm these observations and better inform clinical decision-making. Overall, it remains challenging to identify which patients are at the highest conversion to subsequent TKA after nonoperative treatment, although the potential benefits of IA-TA-ER should continue to be explored.

## Funding

Primary funder was Pacira BIosciences, Inc.

## Conflicts of interest

Andrew L. Concoff is a paid employee for Exagen Inc.; is a paid consultant for United Rheumatology and Pacira BioSciences Inc.; and holds stock or stock options in Exagen Inc. Jennifer H. Lin is a paid employee for Pacira BioSciences, Inc. and holds stock or stock options in Pacira BioSciences, Inc. Andrew I. Spitzer received speakers bureau/paid presentations for Pacira and DePuy; is a paid consultant for Pacira and DePuy; and received research support from Pacira and DePuy as a Principal Investigator. Vinod Dasa received speakers bureau/paid presentations for Bioventus, Pacira, Sanofi, and Sanara; is a paid consultant for Bioventus, Pacira, Sanofi, Ferring, Medi Post, Vertex, Cartiheal, Avania, Nanochon, and Anika; holds stock or stock options in DOC SOCIAL, Goldfinch consulting, Motive, MEND, Grand Care, and Doron Therapeutics; received other financial or material support from My Medical Images, J of Ortho Exp and Innovation, MEND, and Grand Care; and is in the medical/orthopaedic publications editorial/governing board of JOEI. Adam Rivadeneyra. David Rogenmoser. Mitchell K. Ng is a paid consultant for Ferghana Partners, Inc. and Pacira BioSciences, Inc. Mary DiGiorgi is a paid employee for Pacira BioSciences, Inc. and holds stock or stock options in Pacira BioSciences, Inc. Stan Dysart is a paid employee for Pacira BioSciences, Inc. and holds stock or stock options in Pacira BioSciences, Inc. Joshua Urban received speakers bureau/paid presentations from Pacira; is a paid consultant for Pacira and Vertex; holds stock or stock options in Pacira; and received research support from Pacira, Vertex, and SpineBiopharma. William M. Mihalko received royalties from Aesculap Inc.: Vega Knee system and CoreHip femoral stem system; received speakers bureau/paid presentations for Pacira BioSciences; is a paid consultant for Pacira BioSciences; holds stock or stock options in Medtronic Inc.; received research support from Medacta Inc. as a Principal Investigator; received royalties, financial or material support from Elsevier Inc.; is in the medical/orthopaedic publications editorial/governing board of Journal of Arthroplasty, Journal of Orthopaedic Research, J Long Term Effects of Medical Implants, and Orthop Clinics N Am; and is a board member/committee appointments for Knee Society, AAHKS, Hip Society, and ASTM International. Michael A. Mont received royalties from Microport and Stryker; is a paid consultant for DJ Orthopaedics, Johnson and Johnson, Medical Compression Systems, Merz, Orthosensor, Pacira, Sage Products, Inc., Stryker, Tissue Gene, and US Medical Innovations; received research support from DJ Orthopaedics, Johnson and Johnson, National Institutes of Health (NIAMS and NICHD), Ongoing Care Solutions, Orthosensor, Stryker, and Tissue Gene as a Principal Investigator; is in the medical/orthopaedic publications editorial/governing board of Journal of Arthroplasty, Journal of Knee Surgery, Orthopedics, and Surgical Technology International; and is a board member in the American Academy of Orthopaedic Surgeons. All other authors declare no potential conflicts of interest.

For full disclosure statements refer to https://doi.org/10.1016/j.artd.2025.101643.

## CRediT authorship contribution statement

**Andrew L. Concoff:** Writing – review & editing, Supervision, Software, Resources, Formal analysis. **Jennifer H. Lin:** Writing – review & editing, Resources, Project administration, Methodology, Investigation, Formal analysis, Data curation. **Andrew I. Spitzer:** Writing – review & editing, Supervision, Project administration, Methodology, Investigation, Formal analysis. **Vinod Dasa:** Writing – review & editing, Visualization, Project administration, Methodology, Investigation, Formal analysis. **Adam Rivadeneyra:** Writing – review & editing, Visualization, Software, Resources, Methodology, Formal analysis, Data curation. **David Rogenmoser:** Writing – review & editing, Validation, Supervision, Methodology, Investigation, Funding acquisition, Data curation. **Mitchell K. Ng:** Writing – review & editing, Writing – original draft, Validation, Software, Resources, Project administration, Methodology, Investigation, Funding acquisition, Formal analysis, Data curation, Conceptualization. **Mary DiGiorgi:** Writing – review & editing, Visualization, Validation, Supervision, Software, Resources, Project administration, Methodology. **Stan Dysart:** Writing – review & editing, Validation, Supervision, Software, Resources, Project administration, Methodology. **Joshua Urban:** Writing – review & editing, Validation, Supervision, Project administration, Methodology, Investigation, Formal analysis. **William M. Mihalko:** Writing – review & editing, Supervision, Software, Project administration, Methodology, Investigation, Formal analysis. **Michael A. Mont:** Writing – review & editing, Visualization, Validation, Supervision, Methodology, Investigation, Funding acquisition, Conceptualization.

## Appendix A Supplementary data

Conflict of Interest Statement for Concoff

Conflict of Interest Statement for Dasa

Conflict of Interest Statement for DiGeorgi

Conflict of Interest Statement for DySart

Conflict of Interest Statement for Lin

Conflict of Interest Statement for Mihalko

Conflict of Interest Statement for Ng

Conflict of Interest Statement for Rivadeneyra

Conflict of Interest Statement for Rogenmoser

Conflict of Interest Statement for Spitzer

Conflict of Interest Statement for Urban

Conflict of Interest Statement for Mont
