# Supplementary material for: Association of Knee Osteoarthritis Treatment Types, Patient Characteristics, and Medical History With Subsequent Risk for Total Knee Arthroplasty: Data From a New Real-World Registry
Source: Arthroplast Today. 2025 Feb 26;32:101643. doi: 10.1016/j.artd.2025.101643 (PMC11909438; doi:10.1016/j.artd.2025.101643)
Supplement: Conflict of Interest Statement for DiGeorgi [file mmc3.docx]

# INDIVIDUAL CONFLICT OF INTEREST STATEMENT

***American Association of Hip and Knee Surgeons***

(Adopted from the American Academy of Orthopaedic Surgeons disclosure statement)

The following form **must be filled out completely and submitted by each author (example, 6 authors, 6 forms).**

**All items require a response. If there is no relevant disclosure for a given item, enter "*None*.”**

**Manuscript Title: Association of Knee Osteoarthritis Treatment Types, Patient Characteristics, and Medical History with Subsequent Risk for Total Knee Arthroplasty: Data from A New Real-World Registry**

**Manuscript Title**

1. Royalties from a company or supplier (The following conflicts were disclosed)

NONE

2. Speakers bureau/paid presentations for a company or supplier (The following conflicts were disclosed)

NONE

3A. Paid employee for a company or supplier (The following conflicts were disclosed)

Pacira Biosciences, Inc.

3B. Paid consultant for a company or supplier (The following conflicts were disclosed)

NONE

3C. Unpaid consultants for a company or supplier (The following conflicts were disclosed)

NONE

4. Stock or stock options in a company or supplier (The following conflicts were disclosed)

Pacira Biosciences, Inc.

5. Research support from a company or supplier as a Principal Investigator (The following conflicts were disclosed)

NONE

6. Other financial or material support from a company or supplier (The following conflicts were disclosed)

NONE

7. Royalties, financial or material support from publishers (The following conflicts were disclosed)

NONE

8. Medical/Orthopaedic publications editorial/governing board (The following conflicts were disclosed)

NONE

9. Board member/committee appointments for a society (The following conflicts were disclosed)

NONE

**Each author must sign AND print or type his/her name, date and submit a separate form**

In addition, one BLINDED Conflict of Interest form (no author names used) should be submitted per manuscript with all author disclosures.

Mary DiGiorgi, PhD Mary DiGiorgi 4/16/24

Author Name (Print or Type) Author Signature Date
