# Supplementary material for: Association of Knee Osteoarthritis Treatment Types, Patient Characteristics, and Medical History With Subsequent Risk for Total Knee Arthroplasty: Data From a New Real-World Registry
Source: Arthroplast Today. 2025 Feb 26;32:101643. doi: 10.1016/j.artd.2025.101643 (PMC11909438; doi:10.1016/j.artd.2025.101643)
Supplement: Conflict of Interest Statement for Mont [file mmc12.docx]

# INDIVIDUAL CONFLICT OF INTEREST STATEMENT

***American Association of Hip and Knee Surgeons***

(Adopted from the American Academy of Orthopaedic Surgeons disclosure statement)

The following form **must be filled out completely and submitted by each author (example, 6 authors, 6 forms).**

**All items require a response. If there is no relevant disclosure for a given item, enter "*None*.”**

**Manuscript Title: Association of Knee Osteoarthritis Treatment Types, Patient Characteristics, and Medical History with Subsequent Risk for Total Knee Arthroplasty: Data from A New Real-World Registry**

**Manuscript Title**

1. Royalties from a company or supplier (The following conflicts were disclosed)

Microport, Stryker

2. Speakers bureau/paid presentations for a company or supplier (The following conflicts were disclosed)

NONE

3A. Paid employee for a company or supplier (The following conflicts were disclosed)

NONE

3B. Paid consultant for a company or supplier (The following conflicts were disclosed)

DJ Orthopaedics, Johnson and Johnson, Medical Compression Systems, Merz, Orthosensor, Pacira, Sage Products, Inc., Stryker, Tissue Gene, US Medical Innovations

3C. Unpaid consultants for a company or supplier (The following conflicts were disclosed)

NONE

4. Stock or stock options in a company or supplier (The following conflicts were disclosed)

NONE

5. Research support from a company or supplier as a Principal Investigator (The following conflicts were disclosed)

DJ Orthopaedics, Johnson and Johnson, National Institutes of Health (NIAMS and NICHD), Ongoing Care Solutions, Orthosensor, Stryker, Tissue Gene

6. Other financial or material support from a company or supplier (The following conflicts were disclosed)

NONE

7. Royalties, financial or material support from publishers (The following conflicts were disclosed)

NONE

8. Medical/Orthopaedic publications editorial/governing board (The following conflicts were disclosed)

Journal of Arthroplasty, Journal of Knee Surgery, Orthopedics, Surgical Technology International

9. Board member/committee appointments for a society (The following conflicts were disclosed)

American Academy of Orthopaedic Surgeons

**Each author must sign AND print or type his/her name, date and submit a separate form**

In addition, one BLINDED Conflict of Interest form (no author names used) should be submitted per manuscript with all author disclosures.

Michael A. Mont Michael A. Mont 5/28/24

Author Name (Print or Type) Author Signature Date
